# Comprehensive genomic analysis of primary bone sarcomas reveals different genetic patterns compared with soft tissue sarcomas

**DOI:** 10.3389/fonc.2023.1173275

**Published:** 2023-07-21

**Authors:** Qing Zhang, Yongkun Yang, Xia You, Yongzhi Ju, Qin Zhang, Tingting Sun, Weifeng Liu

**Affiliations:** ^1^ Department of Orthopaedic Oncology, Beijing Ji Shui Tan Hospital, Peking University, Beijing, China; ^2^ The Medical Department, Jiangsu Simcere Diagnostics Co., Ltd, Nanjing, Jiangsu, China; ^3^ Nanjing Simcere Medical Laboratory Science Co., Ltd, Nanjing, Jiangsu, China; ^4^ The State Key Lab of Translational Medicine and Innovative Drug Development, Jiangsu Simcere Diagnostics Co., Ltd, Nanjing, Jiangsu, China

**Keywords:** sarcoma, mutation profile, microsatellite instability, therapeutic gene alteration, tumor mutation burden, PD-L1

## Abstract

**Introduction:**

Sarcomas are classified into two types, bone sarcoma and soft tissue sarcoma (STS), which account for approximately 1% of adult solid malignancies and 20% of pediatric solid malignancies. There exist more than 50 subtypes within the two types of sarcoma. Each subtype is highly diverse and characterized by significant variations in morphology and phenotypes. Understanding tumor molecular genetics is helpful in improving the diagnostic accuracy of tumors that have been difficult to classify based on morphology alone or that have overlapping morphological features. The different molecular characteristics of bone sarcoma and STS in China remain poorly understood. Therefore, this study aimed to analyze genomic landscapes and actionable genomic alterations (GAs) as well as tumor mutational burden (TMB), microsatellite instability (MSI), and programmed death ligand-1 (PD-L1) expression among Chinese individuals diagnosed with primary bone sarcomas and STS.

**Methods:**

This retrospective study included 145 patients with primary bone sarcomas (n = 75) and STS (n = 70), who were categorized based on the 2020 World Health Organization classification system.

**Results:**

Patients diagnosed with bone sarcomas were significantly younger than those diagnosed with STS (p < 0.01). The top 10 frequently altered genes in bone sarcoma and STS were TP53, CDKN2A, CDKN2B, MAP3K1, LRP1B, MDM2, RB1, PTEN, MYC, and CDK4.The EWSR1 fusions exhibited statistically significant differences (p < 0.01) between primary bone sarcoma and STS in terms of their altered genes. Based on the actionable genes defined by OncoKB, actionable GAs was found in 30.7% (23/75) of the patients with bone sarcomas and 35.7% (25/70) of those with STS. There were 4.0% (3/75) patients with bone sarcoma and 4.3% (3/70) patients with STS exhibited high tumor mutational burden (TMB-H) (TMB ≥ 10). There was only one patient with STS exhibited MSI-L, while the remaining cases were microsatellite stable. The positive rate of PD-L1 expression was slightly higher in STS (35.2%) than in bone sarcoma (33.3%), however, this difference did not reach statistical significance. The expression of PD-L1 in STS patients was associated with a poorer prognosis (p = 0.007). Patients with STS had a better prognosis than those with bone sarcoma, but the observed difference did not attain statistical significance (p = 0.21). Amplification of MET and MYC genes were negatively correlated with clinical prognosis in bone tumors (p<0.01).

**Discussion:**

In conclusion, bone sarcoma and STS have significantly different clinical and molecular characteristics, suggesting that it is vital to diagnose accurately for clinical treatment. Additionally, comprehensive genetic landscape can provide novel treatment perspectives for primary bone sarcoma and STS. Taking TMB, MSI, PD-L1 expression, and OncoKB definition together into consideration, there are still many patients who have the potential to respond to targeted therapy or immunotherapy.

## 1 Introduction

Most sarcomas arise from the aberrant differentiation process of mesenchymal stem cells and their derived cell lineages. They account for less than 1% of all human cancers but 15%–20% of solid sarcomas in children and adolescents, making them an important group of secondary malignancies (1). Bone and soft tissue sarcomas include primary malignant bone tumors and soft tissue sarcomas (STS) based on anatomical location (2, 3), both of which originate from the mesenchymal system. However, there are many differences between them. First, the subtypes of bone tumors are relatively few, while the subtypes of soft tissue sarcomas are very large. The three main subtypes of adult bone sarcomas are chondrosarcoma (CS) (40%), osteosarcoma (OS) (28%), and chordoma (10%). In contrast, STS account for 70%–80% of all sarcomas, with over 70 different histological subtypes, and each subtype has a different clinical behavior. Among all STS, liposarcoma, leiomyosarcoma, and undifferentiated pleomorphic sarcoma (UPS) are the most common histopathological subtypes. Second, bone and soft tissue sarcomas are strongly associated with age. Patients with bone tumors are younger than those with soft tissue sarcomas. For example, osteosarcoma mainly occurs in teenagers aged 10-20 years. Other osteosarcoma and Ewing’s sarcoma also have obvious age characteristics, and the onset age is younger. With the exception of a few cases in children and adolescents, the majority of soft tissue sarcomas occur in the late 40s and 50s. The most common subtypes, such as pleomorphic undifferentiated sarcoma, liposarcoma, fibrosarcoma, and chondrosarcoma, also tend to occur in middle-aged and elderly patients. Third, there are some clear differences in the early manifestations of bone and soft tissue sarcomas. The most common clinical manifestations of osteosarcoma and Ewing’s sarcoma for bone tumors are painful lumps around the bones and joints, particularly the limbs and knee joints, and this pain tends to worsen, notably intermittent pain at night, which will be very obvious in the later stage. In addition to pain, another major manifestation is progressive mass enlargement. Osteosarcoma is mainly around the joint, and Ewing’s sarcoma is mainly in the middle of the long bone. The onset of soft tissue tumors is more subtle. It usually appears as a painless lump. In particular, some positions are relatively deep in some soft tissue sarcomas, and the tumor was more than 5 centimeters when discovered. At last, there are differences in treatment plans. Although surgery is the primary treatment for sarcoma, since bone tumors affect the movement function of patients, bone and joint reconstruction is also involved in bone tumor resection to help restore movement function. Besides, the effect of chemotherapy for bone tumors is very good, especially the application of neoadjuvant chemotherapy, which can make about 70% of patients survive for five years. While soft tissue sarcomas are much less sensitive to conventional chemotherapy than bone tumors, there have been relatively more advances in targeted and immunotherapy.

Although there is significant histological, genetic, and epigenetic heterogeneity between bone sarcomas and STS, little research has been conducted to determine whether this difference is related to clinical prognosis. In addition to surgery, chemotherapy, and radiotherapy, targeted therapy and immunotherapy are being studied to broaden patients’ treatment options. In this study, we aim to study the differences in histological characteristics, genetic changes, access to targeted drugs, immune biomarkers, and overall prognosis between 75 patients with bone sarcoma and 70 patients with STS by using next-generation sequencing (NGS) technology in conjunction with programmed death ligand-1 (PD-L1) immunohistochemistry testing.

## 2 Methods and materials

### 2.1 Patients

The initial cohort for screening included 339 patients with sarcomas diagnosed between March 2018 and December 2021, and 285 of whom received surgery at participating hospitals (Beijing Ji Shui Tan Hospital). Surgical biopsy samples from 75 patients with primary bone tumors and 70 patients with soft tissue tumors were examined in this study. Finally, the patients provided tumor samples with tumor cells occupying 50% of the content of the tumor tissue were included. All patients had histological diagnosis of solid tumor (regardless of treatment performed). For those patients, the formalin-fixed, paraffin-embedded (FFPE) or fresh samples of sarcomas were available. To detect the genomic aberrations and tumor heterogeneity, all specimens were utilized for more than 500 gene panel DNA next generation sequencing. A series of somatic mutations and copy number variants (CNV) were identified by bioinformatics analysis, including single-nucleotide variants (SNVs)/insertions and deletions (InDels), copy-number variations (CNVs), and structural variations (SVs), was described and compared between primary bone tumors and bone metastases. Finally, the immunogenicity of each tumor region was evaluated, including the tumor mutation numbers, PD-L1 expression, and microsatellite instability status. The present investigation received approval from the ethics committees of the center and was carried out in compliance with the CIOMS guidelines (ethical authorization number: K2022164-00). Furthermore, all participants who were enrolled in the study provided their informed consent by signing the appropriate documentation.

### 2.2 DNA extraction and library preparation

Three commercial kits were used for DNA extraction. Genomic DNA (gDNA) of formalin-fixed and paraffin-embedded (FFPE) tissues and fresh tissues was extracted using the Tissue sample DNA extraction kit (Kai Shuo). Genomic DNA of leucocytes was extracted using MagMAXTM DNA Multi-Sample Ultra Kit (Thermo). Cell-free DNA (cfDNA) of plasma was extracted using MagMAXTM Cell-Free DNA Isolation Kit (Thermo). All of the extraction procedures were performed following the manufacturer’s instructions. DNA was quantified on Qubit Fluorometer with Qubit dsDNA HS Assay kit (Thermo) and its quality was evaluated by Agilent 4200 TapeStation (Agilent).

The probe hybridization capture method was used for library construction. Commercial reagents and customized probes were used for library construction and hybridization capture. In brief, 15 ng-200 ng gDNA was sheared into 200~350 bp by fragmentation enzymes. Indexed paired-end adaptors for the Illumina platform were self-developed and customized (SimcereDx). End repair, A-tailing, and adaptor ligation of sheared DNA and cfDNA was respectively performed by KAPA HyperPlus DNA Library Prep kit (Roche Diagnostics) and VAHTSTM Universal DNA Library Prep Kit for Illumina^®^ (Vazyme). Unligated adaptors were removed by the size selection function of Agencourt AMPure XP beads (Beckman Coulter). The ligation products were PCR amplified to form a pre-library for hybridization. The final library was quantified on Qubit Fluorometer with Qubit dsDNA HS Assay kit (Thermo Fisher) and its quality was evaluated by Agilent 4200 TapeStation (Agilent).

### 2.3 Sequence data processing

The qualified DNA libraries were sequenced on the Illumina NovaSeq6000 platform (Illumina, San Diego, CA) and generated 150 bp paired-end reads. Base calls from Illumina NovaSeq6000 were conducted to FASTQ files. The software fastp (v.2.20.0) was used for adapter trimming and filtering of low-quality bases (4). The BWA-MEM (v.0.7.17) algorithm was performed to align to the reference genome (UCSC’s hg19 GRCh37) (5). Duplicate reads from PCR were excluded using Dedup with Error Correct. SNVs/InDels were called and annotated via VarDict (v.1.5.7) (6) and InterVar (7), then the variants were filtered against the common SNPs in the public database including 1000 Genome Project (Aug 2015) and Exome Aggregation Consortium (ExAC) Browser28 (v.0.3). CNVs and fusions were analyzed by CNV kit (dx1.1) (8) and factera (v1.4.4) (9), respectively.

TMB was defined as the number of somatic, coding, base substitution, and indel mutations per megabase of genome examined. The 539-cancer-gene targeted NGS panel TMB was counted by summing all base substitutions and indels in the coding region of targeted genes, excluding synonymous alterations, alterations of AF < 0.02 and alterations listed as known somatic alterations in COSMIC. The bTMB calculation is the same as the tTMB method, excluding alterations of AF < 0.005.

To determine microsatellite instability (MSI) status, 334 homopolymer repeat loci with adequate coverage on the panel were selected, and reads that were successfully mapped to each of the 334 loci were extracted from the de-duplicated BAM file. Msisensor (10) was employed to evaluate the distribution of read counts among various repeat length and determine the stability of each locus. A MSI score was defined as the percentage of unstable loci. Any sample with a MSI score of ≥0.15 was classified as MSI-H (MicroSatellite Instability-High), and MSI score of ≥0.05 and <0.15 was classified as MSI-L MicroSatellite Instability-Low), otherwise MSS (MicroSatellite Stable).

### 2.4 PD-L1 IHC testing

Take one FFPE slide for each sample for HE staining. After the sample is stained with HE, the pathologist will evaluate the tumor cells in the sample, and exclude unqualified samples with less than 100 tumor cells. The PD-L1 (SP263) experiment was performed on the BenchMark ULTRA automatic immunohistochemical instrument. The experimental steps are as follows: put the slices into the incubator and bake the slices, incubate at 65°C for 30-60min, and pretreat the samples. Next, according to VENTANA official setting procedure, the machine was loaded to complete the IHC automatic dyeing experiment scheme. Finally, the pathologist reviewed the stained sections and gave PD-L1 staining results.

### 2.5 Statistical analysis

The Kaplan-Meier curve analysis overall survival was compared using the log-rank test. All reported P values were two-tailed, and P < 0.05 was considered statistically significant. Statistical analyses were performed using R v. 4.0.3 (https://www.r-project.org).

## 3 Results

### 3.1 Basic characteristics of clinical samples

The detailed clinical and pathological characteristics of the 75 patients with bone sarcoma and 70 patients with STS are summarized in **Supplementary Tables 1** and **2**. All patients were retrospectively enrolled and agreed to undergo NGS of tumor tissues and paired germline samples. PD-L1 expression was detected in 82.8% (n = 120) of the patients.

**Figure 1:**
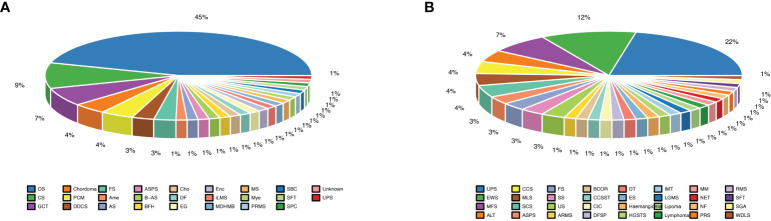
Different subtypes of bone and soft tissue sarcomas. **(A)** Almost half of bone sarcomas are osteosarcomas; **(B)** Undifferentiated pleomorphic sarcoma subtype was the largest proportion of soft tissue sarcomas.

Most of the patients with primary bone sarcomas (48.0%) were in stage II, 33.3% in stage III, and 14.7% in stage I, and the other was unknown. There were 25 pathological subtypes in primary bone sarcomas, most of which were OS (n = 34); the other subtypes included CS (n = 7), giant cell tumor of bone (GCT, n = 5), and other subtypes with fewer than 3 cases in each subtype (**Figure 1A**, **Supplementary Table 1**). Most of the patients with STS (57.1%) were in stage II, 27.1% in stage III, and 8.6% in stage I, and the other was unknown. STS contained 32 pathological subtypes in total, most of which were UPS (n = 15), followed by EWS (n = 8) and MFS (n = 5), with other types being less than 3 in each subtype (**Figure 1B**, **Supplementary Table 2**). Patients with primary bone sarcoma were significantly younger than those with STS, with a median age of 24 (range: 8–78) and 52 (range: 9–83), respectively (p < 0.01, **Table 1**).

**Table 1:** Comparison of clinical features between bone sarcoma and soft tissue sarcoma patients.

Characteristics	Bone sarcomas	Soft tissue sarcomas	*P* value	Method
**Age**	24 (8-78)	52 (9-83)	<0.01	Wilcoxon
**Sex**			0.72	chisq.test
Female	30 (40.00%)	26 (37.14%)		
Male	45 (60.00%)	44 (62.86%)		
**Stage**			0.42	chisq.test
**I**	11 (14.67%)	6 (8.57%)		
**II**	36 (48.00%)	40 (57.14%)		
**III**	25 (33.33%)	19 (27.14%)		
**Unknown**	3 (4%)	3 (4%)		

### 3.2 Differentially mutated genes between primary bone sarcomas and STS

**Figure 2:**
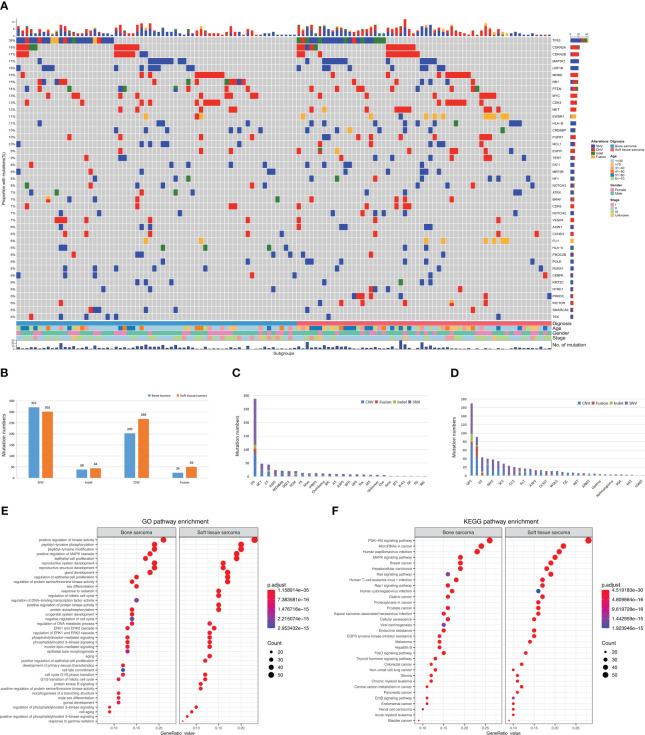
Landscape and distribution differences of genomic alterations in bone and soft tissue sarcomas. **(A)** Mutational landscape of bone and soft tissue sarcomas. Each row represents a gene and each column represents a patient. The mutational spectrum is grouped according to the histological subtype and previous lines of therapies of each patient. On top of the mutation heatmap, the histogram represents the number of mutations per patient. The mutation frequency of each gene is shown on the right side of the mutation heatmap. SNV, single-nucleotide variants; CNV, copy-number variations; InDel, insertion or deletion. **(B)** Comparison of four genomic alterations between bone and soft tissue sarcomas. **(C)** Genomic alterations in bone sarcomas. **(D)** Genomic alterations in soft tissue sarcomas. **(E)** GO analysis of mutations. **(F)** KEGG analysis of mutations.

Mutational profiles and genomic alterations (GAs) of bone sarcomas and STS are shown in **Figure 2A**. A total of 663 somatic alterations containing SNVs, InDels, CNVs, and fusions in STS were identified, which were significantly more than bone sarcomas (n = 586, p = 0.002, **Figure 2B**). The median number of alterations was 6 (range: 1–26) in bone sarcomas and 6 (range: 1–40) in STS. Somatic SNVs and CNVs were the most common mutations between bone sarcomas and STS. The GAs of different pathological subtypes is summarized in **Supplementary Tables 3** and **4**, with OS and UPS having the maximum alterations respectively (**Figures 2C, D**).

Similar GAs was discovered in bone sarcomas and STS, with mutation frequencies of TP53, MAP3K1, LRP1B, RB1, PTEN, and VEGFA greater than 10%. CNVs mainly occurred in CDKN2A, CDKN2B, CDK4, MYC, MDM2, MET, and FGFR1 genes. Moreover, differences in GAs were observed between bone sarcomas and STS. In bone sarcomas, four types of mutations (InDel, CNV, SNV, and fusion) were found in TP53, but no fusions were found in STS. Furthermore, STS had a higher mutation frequency of EWSR1 fusions than bone sarcomas (p < 0.01).

The Kyoto Encyclopedia of Genes and Genomes (KEGG) analysis revealed that the mutation genes in primary bone sarcomas and STS were mostly involved in the same pathways, such as PI3K-Akt, miRNA in tumors, and mitogen-activated protein kinase signaling pathways (**Figure 2E**). Human hepatitis B, viral carcinogenesis, and thyroid hormone signaling pathways were only enriched in bone sarcomas. Pathways of the proteoglycans in tumors were enriched in STS. Gene ontology (GO) analysis revealed that both bone sarcoma- and STS-mutated genes were enriched with positive regulation of kinase activity, gland development, and peptidyl-tyrosine phosphorylation pathways (**Figure 2F**). The DNA-binding transcription factor activity and negative regulation of cell cycle pathways were specifically involved in bone primary sarcomas, whereas the response to radiation was enriched in STS.

### 3.3 Distinct somatic mutation profile between Chinese sarcomas and Western sarcomas

**Figure 3:**
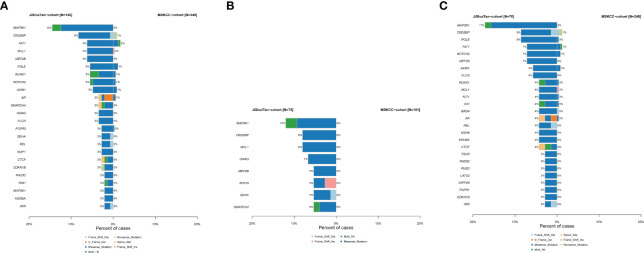
The difference of genomic mutations in sarcomas. We compared the data from JiShuiTan cohort with the sarcoma dataset from MSKCC cohort. **(A)** All sarcomas, **(B)** bone sarcomas, and **(C)** STS.

To further understand whether the somatic mutation profile of the Chinese sarcoma patients is ethnically distinct, we compared the data from our cohort (JiShuiTan cohort) with the sarcoma dataset from the Memorial Sloan Kettering Cancer Center (MSKCC) cohort. Our analysis revealed that in the 349 patients of MSKCC dataset, sarcoma patients had significantly less mutations than JiShuiTan cohort in MAP3K1, CREBBP, FAT1, MCL1, MEF2B, POLE, RUNX1, NOTCH2, and AXIN1 (**Figure 3A**). In bone tumors, the MSKCC cohort had significantly fewer mutations in MAP3K1, CREBBP, MCL1, GNAQ, MEF2B, MYCN, SDHA, and SMARCA4 (**Figure 3B**). While in STS, the MSKCC cohort had significantly fewer mutations in MAP3K1, CREBBP, POLE, FAT1, NOTCH2, MEF2B, AXIN, and FLCN (**Figure 3C**).

### 3.4 Actionable GAs and targeted therapies

**Figure 4:**
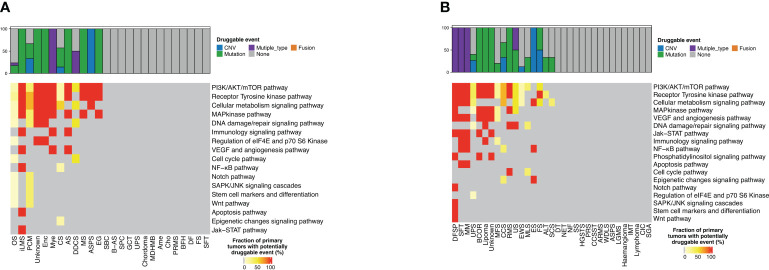
Distribution of targetable genomic alterations in bone sarcomas and soft tissue sarcomas. Proportion of bone sarcomas **(A)** and soft tissue sarcomas **(B)** with potentially druggable events and associated biological pathways, per cancer type.

A total of 31 actionable GAs were detected in 23 patients with bone sarcoma compared with 36 actionable GAs in 25 patients with STS, with the same 12 actionable genes between them. In bone sarcomas, MET CNV (n = 5) was the most frequent actionable GAs, followed by IDH1 SNV (n = 4) and FGFR3 SNV (n = 3). 34.5% (8/23) of the patients with bone sarcomas had two actionable GAs, and 32% of GAs in bone sarcomas were found in OS (n = 10). These genes were found to be enriched in PI3K/AKT/mTOR, receptor tyrosine kinase (RTK), cellular metabolism, map kinase pathway, and DNA damage/repair signaling pathways (**Table 2**, **Figure 4A**).

**Table 2:** Actionable genomic alterations between bone and soft tissue sarcoma patients.

Gene	Bone sarcomas (%)	Soft tissue sarcomas (%)
MET	16.1%	27.8%
IDH1	12.9%	2.8%
FGFR3	9.7%	2.8%
CDK12	6.5%	5.6%
NF1	6.5%	5.6%
TSC2	6.5%	2.8%
ALK	6.5%	0
BRAF	6.5%	0
PIK3CA	3.2%	5.6%
SMARCB1	3.2%	5.6%
BRCA1	3.2%	2.8%
KRAS	3.2%	2.8%
NRAS	3.2%	2.8%
RET	3.2%	2.8%
IDH2	3.2%	0
RAD51D	3.2%	0
TSC1	3.2%	0
EGFR	0	8.3%
BRCA2	0	5.6%
KIT	0	5.6%
ERBB2	0	2.8%
FLT3	0	2.8%
PALB2	0	2.8%
PDGFRA	0	2.8%

As for STS, MET CNV (n = 9) was the most frequent actionable GAs, followed by NF1 InDel and CDK12 SNV, PIK3CA SNV, and KIT SNV (n = 2). 25% (7/25) of the patients with STS had more than one actionable GA, and one of them had four GAs. UPS had the highest percentage of GAs, about 16.7% (n = 6), and those genes were commonly enriched in PI3K/AKT/mTOR, RTK, cellular metabolism, map kinase pathway, and DNA damage/repair signaling pathways. Furthermore, we discovered two GAs in the ALK and BRAF genes that occurred in bone sarcomas, while three GAs in the EGFR gene and one GA in the ERBB2 gene were only found in STS (**Table 2**, **Figure 4B**).

### 3.5 Analysis of tumor mutational burden, PD-L1 expression, and microsatellite status

**Figure 5:**
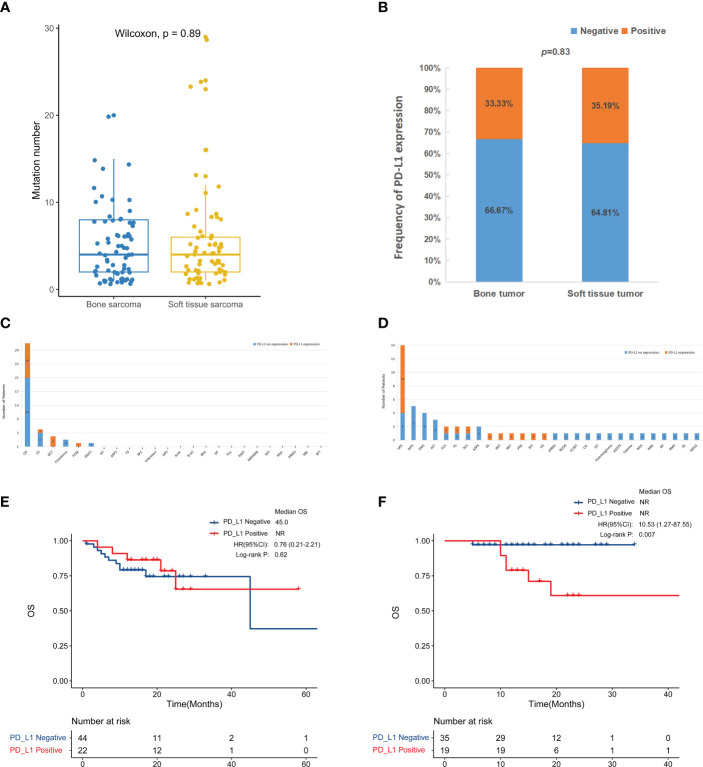
Compare immune-related biomarkers between bone tumors and soft tissue sarcomas. **(A)** Tumor mutation number (p = 0.89). The medium mutation number of bone sarcomas and soft tissue sarcomas was 4 (range: 1-20) and 4 (range: 1-29), respectively. **(B)** PD-L1 positive rate (p = 0.8315). PD-L1 testing in bone sarcomas **(C)** and soft tissue sarcomas **(D)**. Kaplan-Meier survival curves for overall survival in bone sarcomas **(E)** and STS **(F)** according to PD-L1 expression.

The predictive genomic biomarkers for immunotherapy included TMB, MSI, and PD-L1 (11–13). The medium TMB for both bone sarcomas and STS was 4 (**Figure 5A**). There was only one patient with STS with MSI-L, while the others were all microsatellite stable. PD-L1 immunohistochemistry testing was performed in 66 patients with primary bone sarcoma and 54 patients with STS, and the positive rate of PD-L1 expression was 33.3% and 35.2% in bone sarcomas and STS, respectively, with no statistically significant differences (p = 0.83, **Figure 5B**). For bone sarcomas, the top 3 subtypes were OS, CS, and GCT. The positive rate of PD-L1 expression in GCT was the highest (75%, 3/4), followed by OS (32.3%, 10/31) and CS (16.7%, 1/6). Two cases of plasma cell myeloma (PCM) and one case in each group of UPS, Angiosarcoma (AS), ASPS, fibrosarcoma (FS) were positive for PD-L1 expression. The top three STS subtypes were UPS, MFS, and EWS, but only UPS had samples tested positive of PD-L1 expression (71.4%, 10/14). Half of the patients were positive for PD-L1 in clear cell sarcoma (CCS), FS, and spindle cell sarcoma (SCS). The epithelioid sarcoma (ES), inflammatory myofibroblastic tumor (IMT), neuroendocrine tumor (NET), post radiation sarcoma (PRS), solitary fibrous tumor (SFT), and undifferentiated sarcoma (US) had only one patient, and they were all PD-L1 positive (**Figures 5C, D**).

Due to the small number of TMB-H and MSI-H patients, we were not able to perform a more in-depth survival analysis. We further analyzed the survival outcome according to PD-L1 expression. In the bone tumor patients, the association between PD-L1 expression and OS was unclear (**Figure 5E**). While among STS patients, there was a trend toward a worse overall survival for the positive PD-L1 expression group compared to the negative group (p < 0.01) (**Figure 5F**).

### 3.6 Correlation analysis of OS with bone sarcoma and STS

**Figure 6:**
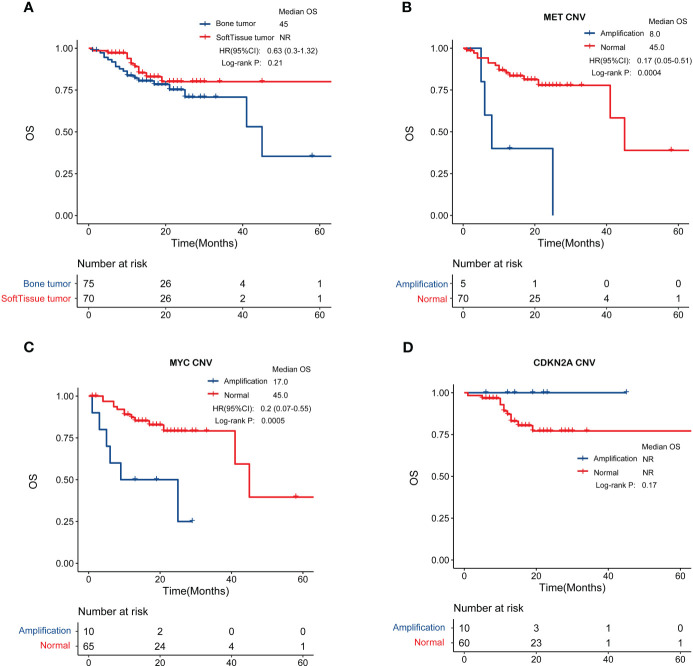
Genomic features in relation to overall survival. **(A)** Patients with bone sarcomas and STS have similar clinical outcomes. **(B, C)** MET and MYC gene amplification was negatively correlated with prognosis in bone sarcomas. **(D)** CDKN2A amplification were associated with better overall survival in STS.

The prognosis of patients with bone sarcoma and STS is poor. In this study, no significant difference in the overall survival of bone sarcomas and STS was observed (p = 0.21, **Figure 6A**).

Through an examination of the correlation between clinicopathological characteristics, including age, sex, and tumor stage, and prognosis, it was determined that there exist variations in overall survival among individuals with bone sarcomas who present with different stages (p < 0.01) (**Supplementary Figure S1A**). The study findings revealed no significant association between the overall survival of STS patients and their age, sex, or stage, as depicted in **Supplementary Figure S1B**.

We also investigated biomarkers associated with patient prognosis by analyzing the relationship between GAs and overall survival. In bone sarcomas, MET amplification (hazard ratio [HR] = 0.17, 95% confidence interval [CI]: 0.05–0.51, p < 0.001) or MYC amplification (HR = 0.2, 95% CI: 0.07–0.55, p < 0.001) were significantly associated with worse overall survival (**Figures 6B, C**). In STS, patients with CDKN2A amplification were associated with better overall survival (**Figure 6D**), though the difference was not statistically significant (p = 0.17).

## 4 Discussion

Sarcomas are highly variable in their biology, and each histologic subtype should be considered a separate therapeutic challenge that requires a distinct understanding of its immune and molecular biology. Several genomic analyses of sarcomas, especially STS, have been published in recent years (14, 15), but the situation in bone sarcomas has not been fully investigated. Although both are classified as sarcomas, bone tumours and soft tissue sarcomas have significant differences in clinical characteristics, malignancy and treatment regimens, so it is necessary to conduct a systematic comparative analysis of them. This research first described the similarities and differences in the molecular landscapes of bone sarcomas and STS.

Although no significant difference was observed in stage or gender distributions, patients with STS were younger than those with bone sarcomas. Even though the TMB, MSI, and PD-L1 expression status appear to follow the same pattern in bone sarcomas and STS, there are some differences in subtypes. The UPS and GCT have a relatively high positive rate of PD-L1 expression in our study. Seth M. Pollack et al. also found that UPS have higher levels of PD-L1 on immunohistochemistry than other STS subtypes (16). This could explain why UPS responded positively to immunotherapy in SARC028 (17). It was identified that neutrophils activation and MHC antigen processing were up-regulated in UPS, which may be beneficial for ICI-based therapy. On the other hand, neutrophil-mediated immunity and immunoglobulin genes were down-regulated in chemotherapy-responsive STS patients (18). Previous studies have reported that systemic immune-inflammation markers could serve as prognostic predictors in tumor ICI-based therapy indicating worse outcomes (19, 20). Fausti et al. confirmed that the high eutrophil-to-lymphocyte ratio (NLR), platelet-to-lymphocyte ratio (PLR) and systemic inflammatory index (SII) were significantly associated with worse progression-free survival (PFS) (p = 0.019; p = 0.004; p = 0.006) in STS patients who received second-line treatment after progressing to anthracycline (21).

Considering the high number of patients with OS and CS, more research into immune checkpoint blockade in OS and CS is needed. Although the PD-L1-positive rate of GCT is very high, patients can achieve a good prognosis through surgical treatment recently, and the immunotherapy in GCT needs more clinical researches. In addition, our results revealed that the PD-L1 expression was not correlated with the prognosis of bone tumours, but negatively correlated with the prognosis of STSs (p=0.007). Chan Kim et al. also showed that STS patients with PD-L1 expression had worse overall survival compared with those without PD-L1 expression (22).

Moreover, there are some significant differences in genetic mutations and their relationship with prognosis between bone sarcomas and STS. For example, EWSR1 and FLT1 fusions, as well as RICTOR amplification, were significantly more common in STS, whereas TP53 fusions and PRKDC alterations were only found in bone sarcomas. In addition, compared with the MSKCC cohort, the JiShuiTan cohort has many unique gene mutations, especially MAP3K1, CREBBP, and MEF2B.

Prognostic analysis did not determine the median survival time. Therefore, the follow-up remained necessary. From our available data, we found that there was no significant difference in OS between bone tumours and soft tissue sarcomas, which was consistent with the trend of previous research literature. In detail, the 5-year survival rate is 67.4% from 2012 to 2018 (23) and the median 5-year survival rate for STS is 65.4% (24–26). However, the associated genetic variants with survival were different between the two groups of patients. The STS showed more heterogeneity than bone sarcomas, and there is no gene significantly associated with prognosis. In contrast, in bone sarcomas, the amplification of MET and MYC was significantly associated with prognosis, and the prognosis of patients with amplification of these two genes was significantly worse than that of patients without amplification. The MET proto-oncogene encodes the hepatocyte growth factor (HGF) receptor MET, which is an RTK involved in carcinogenesis. Aberrant activation of HGF/MET signalling is involved in core oncogenic phenotypes, including uncontrolled cell proliferation, invasion, and metastasis. Interactions between tumour cells and the microenvironment are critical in some cancers and play a central role in multiple myeloma. The network between plasma cells and surrounding cells is also responsible for increased angiogenesis, unbalanced bone formation, and bone lesions. The MET/HGF pathway is a key player in this interaction and is hyperactive in malignant plasma cells and surrounding cells. Patients with abnormal MET and/or HGF levels generally have a poor prognosis (27, 28). MET overexpression was found to be oncogenic in OS and essential for the maintenance of the cancer phenotype (29, 30), and MET inhibitors could inhibit OS growth (31). MET is also involved in CS cell growth regulation (32). Several studies have reported that c-MET is overexpressed in ASPS, and MET inhibitors exert anti-tumour activity in ASPS (33). The results of this study revealed a significantly worse prognosis for patients with MET amplification than that for patients without MET amplification, which is consistent with previous studies. MYC oncogene amplification may play an important role in developing certain STS, as in other human malignancies (34). In patients with OS, high MYC expression is associated with poor survival (35). Additionally, MYC amplifications in OS were significantly correlated with poor event-free survival, independent of the presence of primary metastases (36). MYC amplification can be used as a marker of prognostic importance in chondrosarcoma, associated with a worse prognosis (37). CDKN2A alterations, especially deletions were more common in Ewing sarcoma (38, 39). The CDKN2A deletion has been revealed as a biomarker for poor prognosis in STS (40). And similarly, our study showed that the amplification of CDKN2A was associated with better prognosis in STS.

A limitation of this study was the short follow-up time of patients’ prognosis in the real world, and the survival and prognosis information of patients’ needs to be followed up. Furthermore, the genetic test samples included FFPE and fresh tissue samples, since DNA coming from these two kinds of samples typically greatly differs in terms of quality and integrity, thereby potentially affecting genomic results.

In conclusion, this study describes the detailed genomic landscape and disparity between primary bone sarcomas and STS. The evidence for genetic and immunogenic similarities and identified differences during sarcomas in our study strongly indicates that bone sarcomas and STS should be treated separately, thereby contributing to personalized therapy for patients. In addition to chemotherapy regimens, other targeted and immunotherapy regimens may be considered depending on the patient’s genetic test results.

## 5 Conclusion

This study demonstrates that the implementation of NGS provides a framework for comprehensively interrogating actionable mutations in sarcomas. Comprehensive genomic profiling shows promise to identify targeted therapeutic approaches to improve outcomes for this devastating disease because of the poor prognosis of sarcomas treated by non-targeted conventional therapies, such as chemotherapy, surgery, and irradiation. Considering TMB, MSI, PD-L1 expression, and OncoKB definition together, many patients remain to have the potential to respond to targeted therapy or immunotherapy. However, the main treatments for patients with primary bone sarcomas and STS are rather similar. This study shows significantly different clinical and molecular characteristics between them, suggesting the need to make certain classifications in clinical treatment directions.

## Data availability statement

The datasets used in this article are not accessible because of the Regulations on Management of Human Genetic Resources (Guo Ling No.71) in China. These regulations state that human genetic resource data should not be made publicly available. Requests to access the datasets should be directed to the corresponding author of Qing Zhang, qingzhang_2022@163.com.

## Author contributions

XY and YJ prepared the manuscript and the literature search. QZ collected and analysed the data. TS reviewed and edited the manuscript. QZ and YY treated and observed the patient, prepared samples and gathered detailed clinical information for the study. WL performed the research, collected and analysed the data, interpreted the results and helped revise the manuscript. All authors contributed to the article and approved the submitted version. The authors have read and approved the final manuscript.

## Supplementary material

Supplementary Figure 1 The correlation between clinicopathological characteristics, including age, sex, and tumor stage, and overall survival. **(A)** There exist variations in overall survival among individuals with bone sarcomas who present with different stages (p < 0.01). **(B)** No significant association between the overall survival of STS patients and their age, sex, or stage.

## Glossary

**Table:**

Acinar rhabdomyosarcoma	ARMS
Alveolar soft part sarcoma	ASPS
Ameloblastoma	Ame
Angiosarcoma of bone	B-AS
Angiosarcoma	AS
Atypical lipomatous tumor	ALT
Benign fibrous histiocytoma of bone	BFH
CIC-rearranged sarcoma	CIC
Chondrosarcoma	CS
Chondromatosis	CHO
Clear cell sarcoma	CCS
Clear cell sarcoma of soft tissue	CCSST
Dedifferentiated chondrosarcoma	DDCS
Dedifferentiated adamantinoma	dDA
Desmoplastic fibroma of bone	DF
Desmoid tumor	DT
Dermatofibrosarcoma protuberans	DFSP
Enchondroma	Enc
Eosinophilic granuloma	EG
Ewing sarcoma	EWS
Epithelioid sarcoma	ES
Extraosseous myxoid chondrosarcoma	EMC
Fibrosarcoma	FS
Giant cell tumor of bone	GCT
Hemangioendothelioma	HE
High-grade soft tissue sarcoma	HGSTS
Intraosseous leiomyosarcoma	iLMS
Inflammatory myofibroblastic tumor	IMT
Low-grade myofibroblastic sarcoma	LGMS
Liposarcoma	LPS
Mesenchymal sarcoma	MS
Malignant melanoma	MM
Myeloma	Mye
Myogenic differentiated high-grade malignant bone tumor	MDHMB
Myofibroblastoma	MFB
Myxofibrosarcoma	MFS
Myxoid chondrosarcoma	MC
Myxoid liposarcoma	MLS
Neuroendocrine tumor	NET
Neurofibromas	NF
Osteosarcoma	OS
Osteochondroma	OC, Osteolysis, OL
Plasma cell myeloma	PCM
Postradiation sarcoma	PRS
Pleomorphic rhabdomyosarcoma	PRMS
Rhabdomyosarcoma	RMS
Sarcoma with BCOR genetic alternations	BCOR
Simple bone cyst	SBC
Solitary fibrous tumor	SFT
Spindle cell sarcoma	SCS
Sweat gland adenocarcinoma	SGA
Synovial sarcoma	SS
Undifferentiated sarcoma	US
Undifferentiated pleomorphic sarcoma	UPS
Well-differentiated liposarcoma	WDLS
