# Supplementary figures and images for: Comprehensive genomic analysis of primary bone sarcomas reveals different genetic patterns compared with soft tissue sarcomas
Source: Front Oncol. 2023 Jul 21;13:1173275. doi: 10.3389/fonc.2023.1173275 (PMC10401477; doi:10.3389/fonc.2023.1173275)

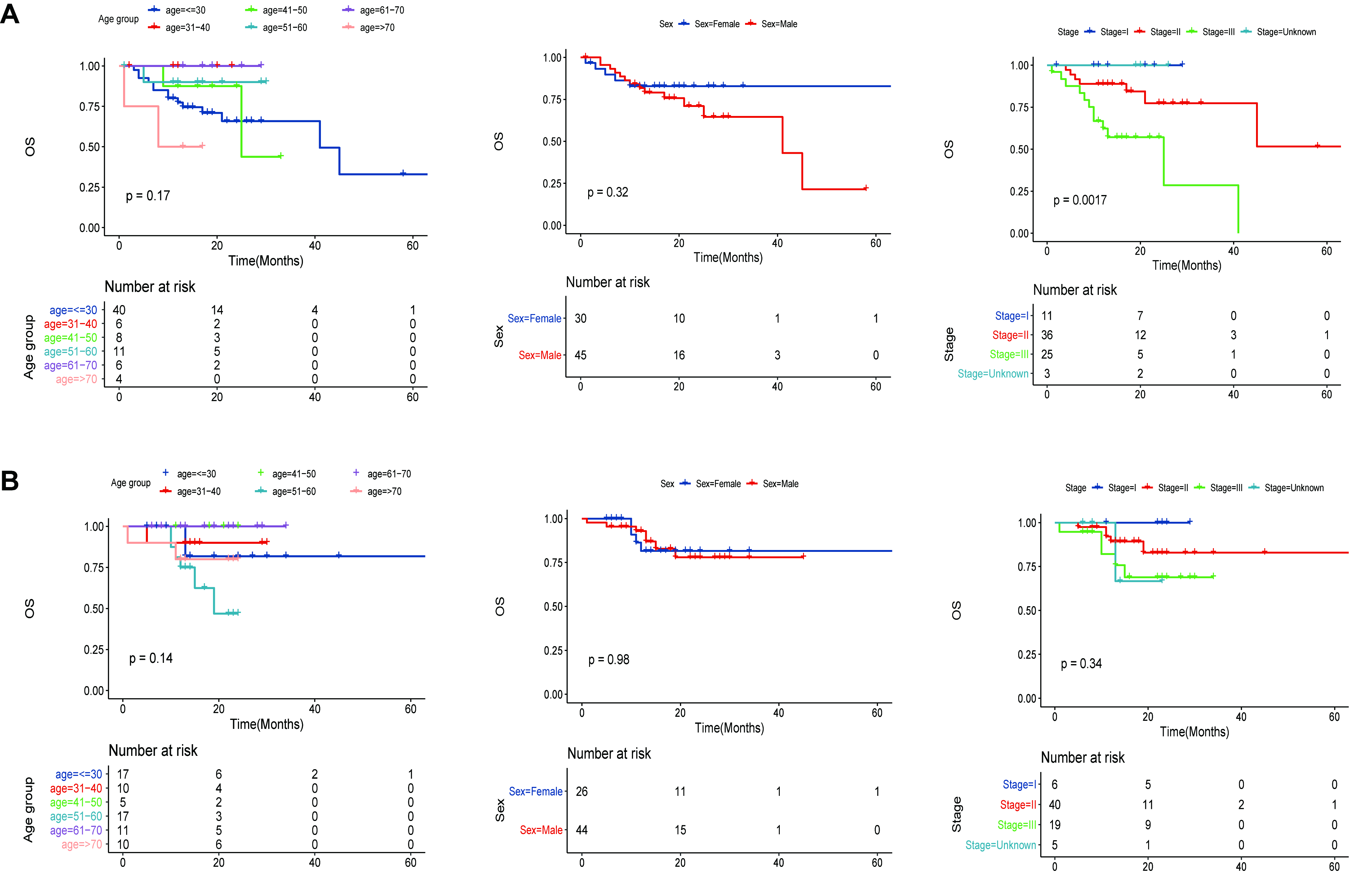

Supplement: Supplementary Figure 1 — The correlation between clinicopathological characteristics, including age, sex, and tumor stage, and overall survival. (A) There exist variations in overall survival among individuals with bone sarcomas who present with different stages (p < 0.01). (B) No significant association between the overall survival of STS patients and their age, sex, or stage. [file Image_1.tif]
