# Supplementary material for: Comprehensive genomic analysis of primary bone sarcomas reveals different genetic patterns compared with soft tissue sarcomas
Source: Front Oncol. 2023 Jul 21;13:1173275. doi: 10.3389/fonc.2023.1173275 (PMC10401477; doi:10.3389/fonc.2023.1173275)
Supplement: Supplementary file 2 [file Table_1.docx]

**Supplementary Table 1** **Summary of clinical and pathologic information in bone sarcomas.**

| **No. of patients** | **75** |
| --- | --- |
| **Age** | 24 (8-78) |
| **Sex** |  |
| Female | 30 (40.0%) |
| Male | 45 (60.0%) |
| **Stage** |  |
| I | 11 (14.7%) |
| II | 36 (48.0%) |
| III | 25 (33.3%) |
| Unknown | 3 (4.0%) |
| **Subtype** |  |
| OS | 34 (45.3%) |
| CS | 7 (9.3%) |
| GCT | 5 (6.7%) |
| Chordoma | 3 (4.0%) |
| PCM | 3 (4.0%) |
| DDCS | 2 (2.7%) |
| FS | 2 (2.7%) |
| Ame | 1 (1.3%) |
| AS | 1 (1.3%) |
| ASPS | 1 (1.3%) |
| B-AS | 1 (1.3%) |
| BFH | 1 (1.3%) |
| Cho | 1 (1.3%) |
| DF | 1 (1.3%) |
| EG | 1 (1.3%) |
| Enc | 1 (1.3%) |
| iLMS | 1 (1.3%) |
| MDHMB | 1 (1.3%) |
| MS | 1 (1.3%) |
| Mye | 1 (1.3%) |
| PRMS | 1 (1.3%) |
| SBC | 1 (1.3%) |
| SFT | 1 (1.3%) |
| SCS | 1 (1.3%) |
| UPS | 1 (1.3%) |
| Unknown | 1 (1.3%) |

OS, Osteosarcoma; CS, Chondrosarcoma; GCT, Giant cell tumor of bone; PCM, Plasma cell myeloma; DDCS, Dedifferentiated chondrosarcoma; FS, Fibrosarcoma; Ame, Ameloblastoma; AS, Angiosarcoma; ASPS, Alveolar soft part sarcoma; B-AS, Angiosarcoma of bone; BFH, Benign fibrous histiocytoma of bone; Cho, Chondromatosis; DF, Desmoplastic fibroma of bone; EG, Eosinophilic granuloma; Enc, Enchondroma; iLMS, Intraosseous leiomyosarcoma; MDHMB, Myogenic differentiated high-grade malignant bone tumor; MS, Mesenchymal sarcoma; Mye, Myeloma; PRMS, Pleomorphic rhabdomyosarcoma; SBC, Simple bone cyst; SFT, Solitary fibrous tumor; SCS, Spindle cell sarcoma; UPS, Undifferentiated pleomorphic sarcoma

**Supplementary Table 2 Summary of clinical and pathologic** **information in soft tissue sarcomas.**

| **No. of patients** | **70** |
| --- | --- |
| **Age** | 52 (9-83) |
| **Sex** |  |
| Female | 26 (37.1%) |
| Male | 44 (62.9%) |
| **Stage** |  |
| I | 6 (8.6%) |
| II | 40 (57.1%) |
| III | 19 (27.1%) |
| **Unknown** | 5 (7.1%) |
| **Subtype** |  |
| UPS | 15 (21.4%) |
| EWS | 8 (11.4%) |
| MFS | 5 (7.1%) |
| ALT | 3 (4.3%) |
| CCS | 3 (4.3%) |
| MLS | 3 (4.3%) |
| SCS | 3 (4.3%) |
| ASPS | 2 (2.9%) |
| FS | 2 (2.9%) |
| SS | 2 (2.9%) |
| US | 2 (2.9%) |
| ARMS | 1 (1.4%) |
| BCOR | 1 (1.4%) |
| CCSST | 1 (1.4%) |
| CIC | 1 (1.4%) |
| DFSP | 1 (1.4%) |
| DT | 1 (1.4%) |
| ES | 1 (1.4%) |
| Haemangioma | 1 (1.4%) |
| HGSTS | 1 (1.4%) |
| IMT | 1 (1.4%) |
| LGMS | 1 (1.4%) |
| Lipoma | 1 (1.4%) |
| Lymphoma | 1 (1.4%) |
| MM | 1 (1.4%) |
| NET | 1 (1.4%) |
| NF | 1 (1.4%) |
| PRS | 1 (1.4%) |
| RMS | 1 (1.4%) |
| SFT | 1 (1.4%) |
| SGA | 1 (1.4%) |
| WDLS | 1 (1.4%) |
| Unknown | 1 (1.4%) |

UPS, Undifferentiated pleomorphic sarcoma; EWS, Ewing sarcoma; MFS, Myxofibrosarcoma; ALT, Atypical lipomatous tumor; CCS, Clear cell sarcoma; MLS, Myxoid liposarcoma; SCS, Spindle cell sarcoma; ASPS, Alveolar soft part sarcoma; FS, Fibrosarcoma; SS, Synovial sarcoma; US, Undifferentiated sarcoma; ARMS, Acinar rhabdomyosarcoma; BCOR, Sarcoma with BCOR genetic alternations; CCSST, Clear cell sarcoma of soft tissue; CIC, CIC-rearranged sarcoma; DFSP, Dermatofibrosarcoma protuberans; DT, Desmoid tumor; ES, Epithelioid sarcoma; HGSTS, High-grade soft tissue sarcoma; IMT, Inflammatory myofibroblastic tumor; LGMS, Low-grade myofibroblastic sarcoma; MM, Malignant melanoma; NET, Neuroendocrine tumor; NF, Neurofibromas; PRS, Postradiation sarcoma; RMS, Rhabdomyosarcoma; SFT, Solitary fibrous tumor; SGA, Sweat gland adenocarcinoma; WDLS, Well-differentiated liposarcoma

# **Supplementary Table 3 Summary of genomic alterations in bone sarcomas.**

| **Subtype** | **InDel (%)** | **SNV (%)** | **CNV (%)** | **Fusion (%)** | **Total** |
| --- | --- | --- | --- | --- | --- |
| OS | 15 (5.2%) | 1.7 (59.0%) | 87 (30.2%) | 16 (5.6%) | 288 |
| GCT | 1 (2.1%) | 22 (46.8%) | 22 (46.8%) | 2 (4.3%) | 47 |
| CS | 7 (15.9%) | 20 (45.5%) | 17 (38.6%) |  | 44 |
| iLMS | 1 (4.2%) | 19 (79.2%) | 4 (16.7%) |  | 24 |
| MDHMB |  | 8 (38.1%) | 13 (61.9%) |  | 21 |
| DDCS | 1 (5.0%) | 13 (65.0%) | 4 (20.0%) | 2 (10.0%) | 20 |
| PCM | 5 (25.0%) | 9 (45.0%) | 6 (30.0%) |  | 20 |
| FS | 1 (7.7%) | 5 (38.5%） | 7 (53.8%) |  | 13 |
| Mye | 1 (8.3%） | 5 (41.7%） | 6 (50.0%) |  | 12 |
| PRMS | 2 (16.7%） | 1 (8.3%） | 9 (75.0%) |  | 12 |
| Chordoma | 2 (18.2%) | 7 (63.6%) | 2 (18.2%) |  | 11 |
| SCS |  | 8 (80.0%) | 2 (20.0%) |  | 10 |
| AS | 1 (11.1%) | 5 (55.6%) | 3 (33.3%) |  | 9 |
| ASPS |  | 1 (12.5%) | 5 (62.5%) | 2 (25.0%) | 8 |
| BFH |  | 2 (25.0%) | 4 (50.0%) | 2 (25.0%) | 8 |
| UPS |  | 6 (75.0%) | 2 (25.0%) |  | 8 |
| Enc |  | 6 (85.7%) | 1 (14.3%) |  | 7 |
| MS |  | 2 (33.3%) | 4 (66.7%) |  | 6 |
| Unknown |  | 6 (100.0%) |  |  | 6 |
| Cho |  | 1 (25.0%) | 3 (75.0%) |  | 4 |
| Ame |  | 2 (100.0%) |  |  | 2 |
| SFT |  |  | 2 (100.0%) |  | 2 |
| B-AS |  | 1 (100.0%) |  |  | 1 |
| DF |  | 1 (100.0%) |  |  | 1 |
| EG |  |  |  |  | 1 |
| SBC |  | 1 (100.0%) |  |  | 1 |
| Total | 38 (6.5%) | 321 (54.8%) | 203 (34.6%) | 24 (4.1%) | 586 |

OS, Osteosarcoma; GCT, Giant cell tumor of bone; CS, Chondrosarcoma; iLMS, Intraosseous leiomyosarcoma; MDHMB, Myogenic differentiated high-grade malignant bone tumor; DDCS, Dedifferentiated chondrosarcoma; PCM, Plasma cell myeloma; FS, Fibrosarcoma; Mye, Myeloma; PRMS, Pleomorphic rhabdomyosarcoma; SCS, Spindle cell sarcoma; AS, Angiosarcoma; ASPS, Alveolar soft part sarcoma; BFH, Benign fibrous histiocytoma of bone; UPS, Undifferentiated pleomorphic sarcoma; Enc, Enchondroma; MS, Mesenchymal sarcoma; Cho, Chondromatosis; Ame, Ameloblastoma; SFT, Solitary fibrous tumor; B-AS, Angiosarcoma of bone; DF, Desmoplastic fibroma of bone; EG, Eosinophilic granuloma; SBC, Simple bone cyst

**Supplementary Table 4 Summary of genomic alterations in soft tissue sarcomas.**

| **Subtype** | **InDel (%)** | **SNV (%)** | **CNV (%)** | **Fusion (%)** | **Total** |
| --- | --- | --- | --- | --- | --- |
| UPS | 20 (11.8%) | 71 (41.8%) | 73 (42.9%) | 6 (3.5%) | 170 |
| EWS | 3 (3.3%) | 19 (20.9%) | 43 (47.3%) | 26 (28.6%) | 91 |
| US | 4 (9.3%) | 33 (76.7%) |  | 6 (14.0%) | 43 |
| FS | 2 (5.1%) | 11 (28.2%) | 26 (66.7%) |  | 39 |
| MFS |  | 23 (60.5%) | 13 (34.2%) | 2 (5.3%) | 38 |
| MM |  | 16 (47.1%) | 18 (52.9%) |  | 34 |
| SCS |  | 15 (53.6%) | 13 (46.4%) |  | 28 |
| RMS |  | 23 (100.0% ) |  |  | 23 |
| CCS |  | 14 (70.0%) | 4 (20.0%) | 2 (10.0%) | 20 |
| DFSP | 3 (16.7%) | 8 (44.4%) | 5 (27.8%) | 2 (11.1%) | 18 |
| ALT | 2 (11.8%) | 7 (41.2%) | 8 (47.1%) |  | 17 |
| SS |  | 5 (31.3%) | 11 (68.8%) |  | 16 |
| ASPS |  | 8 (61.5%) | 3 (23.1%) | 2 (15.4%) | 13 |
| Unknown | 2 (15.4%) | 3 23.1%() | 8 (61.5%) |  | 13 |
| CCSST |  | 5 (45.5%) | 2 (18.2%) | 4 (36.4%) | 11 |
| SFT |  | 5 (45.5%) | 6 (54.5%) |  | 11 |
| WDLS |  | 6 (54.5%) | 5 (45.5%) |  | 11 |
| NF |  | 4 (44.4%) | 5 (55.6%) |  | 9 |
| CIC |  |  | 7 (100.0%) |  | 7 |
| MLS | 1 (14.3%) | 5 (71.4%) | 1 (14.3%) |  | 7 |
| IMT |  | 1 (16.7%) | 5 (83.3%) |  | 6 |
| Lymphoma | 1 (16.7%) | 5 (83.3%) |  |  | 6 |
| ARMS | 2 (40.0%) | 1 (20.0%) | 2 (40.0%) |  | 5 |
| HGSTS |  | 1 (20.0%) | 4 (80.0%) |  | 5 |
| Lipoma | 2 (50.0%) | 1 (25.0%) | 1 (25.0%) |  | 4 |
| ES |  |  | 3 (100.0%) |  | 3 |
| Haemangioma | 1 (33.3%) | 1 (33.3%) | 1 (33.3%) |  | 3 |
| PRS |  | 2 (66.7%) | 1 (33.3%) |  | 3 |
| SGA |  | 3 (100.0%) |  |  | 3 |
| BCOR |  | 2 (100.0%) |  |  | 2 |
| NET | 1(50.0%) | 1 (50.0%) |  |  | 2 |
| DT |  | 1 (100.0%) |  |  | 1 |
| LGMS |  | 1 (100.0%) |  |  | 1 |
| Total | 44 (6.6%) | 301 (45.4%) | 268 (40.4%) | 50 (7.5%) | 663 |

UPS, Undifferentiated pleomorphic sarcoma; EWS, Ewing sarcoma; US, Undifferentiated sarcoma; FS, Fibrosarcoma; MFS, Myxofibrosarcoma; MM, Malignant melanoma; SCS, Spindle cell sarcoma; RMS, Rhabdomyosarcoma; CCS, Clear cell sarcoma; DFSP, Dermatofibrosarcoma protuberans; ALT, Atypical lipomatous tumor; SS, Synovial sarcoma; ASPS, Alveolar soft part sarcoma; CCSST, Clear cell sarcoma of soft tissue; SFT, Solitary fibrous tumor; WDLS, Well-differentiated liposarcoma; NF, Neurofibromas; CIC, CIC-rearranged sarcoma; MLS, Myxoid liposarcoma; IMT, Inflammatory myofibroblastic tumor; ARMS, Acinar rhabdomyosarcoma; HGSTS, High-grade soft tissue sarcoma; ES, Epithelioid sarcoma; PRS, Postradiation sarcoma; SGA, Sweat gland adenocarcinoma; BCOR, Sarcoma with BCOR genetic alternations; NET, Neuroendocrine tumor; DT, Desmoid tumor; LGMS, Low-grade myofibroblastic sarcoma
